# Supplementary material for: Factors associated with seclusion and restraint on admission to forensic psychiatric hospitals: A 10-year retrospective study
Source: PLoS One. 2025 Jul 23;20(7):e0328164. doi: 10.1371/journal.pone.0328164 (PMC12286363; doi:10.1371/journal.pone.0328164)
Supplement: S1 Table — (DOCX) [file pone.0328164.s001.docx]

S1 Table: Demographic, administrative, and clinical characteristics among forensic patients from April 1, 2013 to March 31, 2023

| **Patient Characteristics** | **Total sample N = 7635**  **n (%)** |
| --- | --- |
| **Sociodemographic** |  |
| **Age at admission, M (SD)** | 37.69 (12.76) |
| **Sex** |  |
| Male | 6398 (84) |
| Female | 1231 (16) |
| Other | 6 (0.1) |
| **Immigration, including refugee status** |  |
| Never | 6187 (81) |
| More than 1 year ago | 999 (13) |
| 31 days to 1 year ago | 439 (6) |
| 8 to 30 days ago | 3 (0) |
| In last 3 days | 7 (0.1) |
| **Indigenous self-identification (First Nations, Inuit, Aboriginal)** | 502 (7) |
| **Administrative** |  |
| **Forensic status at assessment** |  |
| Fitness assessment | 1667 (22) |
| Not criminally responsible (NCR) assessment | 2053 (27) |
| Treatment order | 1608 (21) |
| Keep fit order | 40 (1) |
| Warrant of committal — unfit | 183 (2) |
| Warrant of committal — NCR | 1487 (19) |
| Inter-hospital transfer — NCR | 105 (1) |
| Inter-hospital transfer — unfit | 10 (0) |
| Other | 482 (6) |
| **Clinical** |  |
| **Diagnosis** |  |
| Schizophrenia and other psychotic disorder | 5672 (74) |
| Substance use disorder | 3688 (48) |
| Mood or anxiety disorder | 1298 (17) |
| Neurocognitive disorder | 318 (4) |
| Personality disorder | 1352 (18) |
| Neurodevelopmental disorder | 501 (7) |
| **Insight** |  |
| Full | 770 (10) |
| Limited insight | 4823 (63) |
| None | 2042 (27) |
| **Medication adherence** |  |
| Always adherent | 2686 (35) |
| Adherent 80% of time or more | 1456 (19) |
| Adherent less than 80% of time, including failure to purchase prescribed medications | 1645 (22) |
| No medications prescribed | 606 (8) |
| Unknown | 1242 (16) |
| **Elopement attempts/threats** |  |
| Indicator not exhibited in the last 3 days | 7248 (95) |
| Indicator not exhibited in the last 3 days but it is reported to be present | 183 (2) |
| Indicator exhibited 1-2 of the last 3 days | 126 (2) |
| Indicator exhibited daily in the last 3 days | 78 (1) |
| **ABS Scale, M (SD)** | 1.85 (2.75) |
| **Violence Sum, M (SD)** | 4.98 (3.46) |
| **Mania Scale, M (SD)** | 3.33 (4.20) |
| **CPS Scale, M (SD)** | 0.61 (1.04) |

M = Mean, SD = Standard Deviation
