# Supplementary material for: Factors associated with seclusion and restraint on admission to forensic psychiatric hospitals: A 10-year retrospective study
Source: PLoS One. 2025 Jul 23;20(7):e0328164. doi: 10.1371/journal.pone.0328164 (PMC12286363; doi:10.1371/journal.pone.0328164)
Supplement: Attachment — Submitted filename: Response to reviewers.docx [file pone.0328164.s003.docx]

Zelalem Belayneh Muluneh
Academic Editor
PLOS ONE

March 17, 2025

Re: revision for PONE-D-24-34524

Factors associated with seclusion and restraint on admission to forensic psychiatric hospitals: A 10-year retrospective study

Dear Dr. Muluneh,

We would like to express our gratitude to you and the reviewers for the time spent reviewing our paper and providing insightful feedback. Your comments have been invaluable and have greatly contributed to the improvements made in the current version. The authors have thoroughly considered each comment and have made every effort to address them all. We have addressed the lack of availability of the raw data and provided a statement explaining why the data are legally restricted. We improved the clarity of our aim, firmed up the introduction and expanded on the Canadian forensic mental health system. We expanded the methodology section by further including references for the variables used and utilized an alternative analysis approach as suggested. We have now employed a multilevel logistic regression model using a LOGIT link (Generalized Linear Mixed Model – GLMM) to account for the clustering of responses within the 10 hospitals; we hope that the revised manuscript meets your high standards.

Please see our point-by-point responses in the table below.

| Editor |  |
| --- | --- |
| submit your revised manuscript by Mar 13 2025 11:59PM | Submitted on Mar 12 2025 |
| Include:   - A rebuttal letter that responds to each point raised by the academic editor and reviewer(s). You should upload this letter as a separate file labeled 'Response to Reviewers'. - A marked-up copy of your manuscript that highlights changes made to the original version. You should upload this as a - separate file labeled 'Revised Manuscript with Track Changes' - An unmarked version of your revised paper without tracked changes. You should upload this as a separate file labeled 'Manuscript'. | All responses are provided in this table.  We are submitting a marked up copy of the manuscript in which all modifications to the manuscript have been highlighted in blue, called “Revised Manuscript with Track Changes”.  We are also submitting an unmarked revised paper called “Manuscript”. |
| Journal requirements: |  |
| 1. Please ensure that your manuscript meets PLOS ONE's [style requirements](https://journals.plos.org/plosone/s/file?id=wjVg/PLOSOne_formatting_sample_main_body.pd), including those for file naming. The PLOS ONE [style templates](https://journals.plos.org/plosone/s/file?id=ba62/PLOSOne_formatting_sample_title_authors_affiliations.pdf) | We have reviewed the PLOS ONE style requirements and ensured we adhere to them throughout the manuscript. |
| 2. We note that you have indicated that there are restrictions to data sharing for this study. For studies involving human research participant data or other sensitive data, we encourage authors to share de-identified or anonymized data. However, when data cannot be publicly shared for ethical reasons, we allow authors to make their data sets available upon request. For information on unacceptable data access restrictions, please see <http://journals.plos.org/plosone/s/data-availability#loc-unacceptable-data-access-restrictions> | The third-party data utilized in this study is owned by the Canadian Institute for Health Information (CIHI) and is considered confidential. This data was accessed through the Ontario Mental Health Reporting System (OMHRS) and may be available upon request to individuals who meet the pre-specified criteria for confidential access, as outlined by CIHI: <https://www.cihi.ca/en/ontario-mental-health-reporting-system-metadata> . The authors cannot publically share the dataset used in the present study due to a signed data use agreement. |
| Before we proceed with your manuscript, please address the following prompts:  a) If there are ethical or legal restrictions on sharing a de-identified data set, please explain them in detail (e.g., data contain potentially identifying or sensitive patient information, data are owned by a third-party organization, etc.) and who has imposed them (e.g., a Research Ethics Committee or Institutional Review Board, etc.). Please also provide contact information for a data access committee, ethics committee, or other institutional body to which data requests may be sent. | The dataset contains potentially identifying information. The data are provided by a third-party organization, the Ontario Mental Health Reporting System (OMHRS). The research team can only share data with other co-investigators approved by the Waypoint Research Ethics Board. We have now provided a statement explaining why the data are legally restricted. |
| b) If there are no restrictions, please upload the minimal anonymized data set necessary to replicate your study findings to a stable, public repository and provide us with the relevant URLs, DOIs, or accession numbers. Please see <http://www.bmj.com/content/340/bmj.c181.long> for guidelines on how to de-identify and prepare clinical data for publication. For a list of recommended repositories, please see <https://journals.plos.org/plosone/s/recommended-repositories>. You also have the option of uploading the data as Supporting Information files, but we would recommend depositing data directly to a data repository if possible.  Please update your Data Availability statement in the submission form accordingly. |  |
| Reviewers' comments: Reviewer's Responses to Questions  **Comments to the Author** |  |
| The manuscript must describe a technically sound piece of scientific research with data that supports the conclusions. Experiments must have been conducted rigorously, with appropriate controls, replication, and sample sizes. The conclusions must be drawn appropriately based on the data presented.  Reviewer #1: Partly  Reviewer #2: Partly | We have improved the scientific strength of the manuscript by an improved data analytic plan as suggested by the reviewers. |
| 2. Has the statistical analysis been performed appropriately and rigorously?  Reviewer #1: No  Reviewer #2: Yes | We have improved the data analytic plan and analysis as suggested by the reviewers. |
| 3. Have the authors made all data underlying the findings in their manuscript fully available?  The [PLOS Data policy](http://track.editorialmanager.com/CL0/http:%2F%2Fwww.plosone.org%2Fstatic%2Fpolicies.action%23sharing/1/010f0194a67a7789-3f66efc1-c159-422a-920b-8ae5af638290-000000/9agJnohWre6VcpT5nzM6a5qs49w1aCGP0SQuhB7e-MU=195) requires authors to make all data underlying the findings described in their manuscript fully available without restriction, with rare exception (please refer to the Data Availability Statement in the manuscript PDF file). The data should be provided as part of the manuscript or its supporting information, or deposited to a public repository. For example, in addition to summary statistics, the data points behind means, medians and variance measures should be available. If there are restrictions on publicly sharing data—e.g. participant privacy or use of data from a third party—those must be specified.  Reviewer #1: No  Reviewer #2: No | We have now provided a statement explaining why the data are legally restricted. |
| 4. Is the manuscript presented in an intelligible fashion and written in standard English?  PLOS ONE does not copyedit accepted manuscripts, so the language in submitted articles must be clear, correct, and unambiguous. Any typographical or grammatical errors should be corrected at revision, so please note any specific errors here.  Reviewer #1: Yes  Reviewer #2: Yes |  |
| **Reviewer #1**: |  |
| This is an extremely important and highly relevant topic. As the authors have noted, the use of seclusion and restraints in psychiatric settings raises significant concerns regarding the protection of patient rights and the trauma experienced by patients and staff involved in these measures. The longitudinal data used in this analysis is excellent for examining these issues in forensic mental healthcare. I found the article engaging and believe it contributes valuable insights, and I would like to see it published. | Thank you for your kind words. The well-being of patients and staff is dear to our hearts, and we continuously aim to add to evidence-based research in both domains. |
| However, several major revisions are needed prior to publication. Broadly, these revisions include providing a more detailed background and discussion of the literature. This should include a deeper analysis of why policies have been unsuccessful in reducing restraints, and a clearer description of the Canadian forensic psychiatric system for readers unfamiliar with it. There are also significant concerns with the methodological approach, particularly the failure to account for clustering effects given the data's collection from multiple hospitals. Lastly, there are some grammatical and stylistic concerns that should be addressed. | Thank you for the thoughtful suggestions. We are now providing a more detailed background and discussion of the literature, including the Canadian context  We have added some more details on why policies are unsuccessful and on the Canadian forensic system, which we detail in our responses below.  In our methods section we now address the clustering effect by revising our regression model: “To account for the clustering of responses within facilities/hospitals, we employed logistic Generalized Linear Mixed Model (logistic GLMM). This approach allowed us to estimate the strength of the association between coercive measures (seclusion and restraint) and patient-level factors, including sociodemographic, administrative, and clinical factors, accounting for clustering within hospitals. We analyzed seclusion and restraint separately.” |
| **1. Abstract and Introduction** The authors clearly summarize the main research question, identifying factors related to seclusion and restraints in forensic mental health care settings. The authors cite relevant literature, particularly highlighting previous work on coercive measures in psychiatric settings and forensic hospitals. However, the paper would benefit from a deeper discussion. Please consider incorporating an overview of the Canadian forensic hospital system and how individuals enter into the healthcare system. Discuss the rationale for restraints and seclusion, and why policies and practices to reduce restraints have not been uniformly successful. Are these failures system-related issues, staffing problems, patient mix/presentation, environmental challenges, etc.? | Thank you for the meaningful suggestions.  We have added some detail on the debate around why policies fail, balancing causes at the systemic and clinical levels, with the addition of three sources. To keep the manuscript to a reasonable length, however, we have not provided a lengthy discussion of this topic. Instead, we crafted the discussion of this to keep the introduction flowing towards our focus on individual risk. We state, “Critics have argued for enhanced regulation of coercive practices (29). Yet, policies may fail because of a belief that seclusion is necessary for the safety of staff and patients (30) despite evidence to the contrary (31). Failure to reduce the seclusion of restraint has led to recommendations for improved clinical techniques and teamwork (32).”  The Canadian forensic psychiatric system has been described in detail. We state, “In Canada, individuals may be detained in a forensic mental health hospital for two main reasons: 1. For short-term evaluation and treatment to assess their fitness to stand trial or determine if they had a mental disorder at the time of the alleged offense, possibly exempting them from criminal responsibility; and 2. For long-term rehabilitation and reintegration after the court finds them unfit to stand trial (UST) or not criminally responsible due to a mental disorder (NCRMD) (41). While some forensic patients may be admitted to units with minimum security (i.e., open or locked door, limited unaccompanied time off unit), many begin their forensic hospitalization under medium security (i.e., locked door with restricted time off unit) or high security (i.e., perimeter security, locked door, restricted movement within the facility).” |
| **2. Methods** Selection of method: More information on hospital-related factors would provide important context. A major methodological concern is that the data was collected from 10 different forensic psychiatric hospitals in Ontario, yet the authors do not account for the potential clustering of patients within these hospitals. Hospital-related characteristics could significantly influence the use of seclusion and restraints. Did the authors consider alternative statistical methods to account for clustering (e.g., GEE)? These models would account for both patient-level and hospital-level characteristics. | We appreciate this insightful suggestion. We gave considerable thought to an alternative statistical approach. We now include hospital as a contributing factor, finding that hospitals indeed contributed to the multilevel logistic regression models using a LOGIT link (logistic GLMM). |
| **Missing data:** The authors do not provide a rationale for why missingness was not considered a concern. This should be addressed. | We have added this sentence to Data Sources: “The variables used in the study were mandatory reporting requirements for Ontario hospitals and must be completed within 72 hours of admission to a mental health bed. A complete dataset with no missing information was obtained, ensuring that missingness did not pose a concern for data analysis.” |
| **Psychiatric Diagnoses and Substance Abuse:** Substance abuse often co-occurs with other psychiatric conditions and can exacerbate aggressive behaviours, potentially increasing the likelihood of seclusion or restraint. Could the authors discuss the rationale for using only a primary diagnosis and not accounting for dual diagnosis? There is also no discussion of whether patients are screened for substances at admission which could affect their behaviour. If this information was unavailable in the data, the authors should indicate this in the paper. | Thank you for your suggestion. We have changed diagnoses to reflect any diagnosis, not only primary diagnosis, which allows for the recognition that substance abuse often co-occurs with other conditions. We now state: “Up to three diagnoses were documented using DSM-IV (n = 2,501) or DSM-5 (n = 5,134). We recoded all diagnoses to indicate whether each individual had a diagnosis within each of six categories: 1 = schizophrenia spectrum and other psychotic disorders, 2 = substance use disorders, 3 = mood or anxiety disorders, 4 = neurocognitive disorders, 5 = personality disorders, 6 = neurodevelopmental disorders.“ The impact of each primary diagnosis is no longer compared to the ref category, psychotic disorder, instead we investigate each diagnosis as an independent predictor variable.  We did not include screening for substances as this variable was not available in our data set. |
| **4. Results** The figures and tables are generally clear and readable, though there are some discrepancies between the text and the tables, such as missing numbers and percentages in the text (n=%) or the layout was inconsistent. | Thank you for your valuable suggestions regarding missing numbers and inconsistent layout. We have added the missing numbers in the text and improved the design of the tables to ensure consistency. |
| **5. Discussion and Conclusions** The study would benefit from a more detailed discussion of hospital characteristics that could influence the use of coercive measures. Additional context is needed to fully understand the Canadian system, particularly regarding fitness-level factors and how these translate to the wider context. I would also appreciate more discussion in the implications section about how to integrate these findings into practice. For instance, pharmacological treatments are an option, as noted, but this is a population that may be reluctant to take medication. A deeper discussion into possible interventions and their challenges would be valuable. | We have given considerable space in the revised manuscript to discussion of hospital characteristics, including a new paragraph which considers some of the hospital characteristics that could influence use of coercive measures, while providing further context about the Canadian system of forensic hospitals. At the same time, we are concerned to return to the focus of our study on individual risk levels. Thus, we state, “We found a moderate clustering effect of forensic facilities in the adjusted logistic GLMM model for seclusion and a minimal effect in the restraint model. The effect on the seclusion model was not clearly attributable to a single outlier, as one facility (Facility H) was substantially more likely to use seclusion than other hospitals, while another facility used seclusion in the near absence of restraint (Facility D), and another used an atypically high proportion of restraint to seclusion (Facility A). Differences may be due to the level of security within each forensic service, which in our study ranged from minimum security with unlocked units to a high secure building with restricted internal movement, and could affect the need for additional restrictive measures. Patient populations within the hospitals may differ in their level of psychiatric acuity and aggressive behavior, as well as demographic characteristics and legal status, which in turn is related to individual risk for seclusion and restraint. For example, in the Canadian context, some forensic units are designed for persons undergoing assessment and may provide services for a higher proportion of individuals being assessed for their current fitness to stand trial, who are more likely to present with active symptoms of mental illness. Other units serve longer-stay patients found not criminally responsible for offenses and who may be successfully treated for mental illness but remain in the forensic system due to concerns about their risk of reoffending if released to the community. While system-level changes may be needed to implement seclusion and restraint reduction strategies across hospitals, our purpose in the present study was to examine individual-level risk factors that lead to improvements in risk assessment and implementation of individualized interventions to prevent seclusion and restraint.”  In the implications section, we have separated the content into a paragraph focusing on research implications and a new paragraph on clinical implications, in which we delve into how our current findings about clinical, demographic, and administrative risk factors can be integrated into clinical practice. We state, “The present findings provide some evidence to support the practice of individual-level assessments and individualized interventions to reduce the risk of seclusion and restraint. Understanding the factors associated with restraint and seclusion within the first three days of forensic admission allows clinicians to identify risk factors in the crucial time period shortly after a patient is admitted and provide timely interventions to limit the onset of the use of coercive measures. Our findings regarding clinical risk factors suggest valuable interventions among at-risk forensic in-patients, including evidence-based pharmacological and psychosocial treatments. In particular, evidence-based interventions for the prevention of in-hospital aggression are indicated by our findings and may include both violence risk assessment (68) and assessments specifically to identify individuals at risk of experiencing coercive measures (5), along with aggression management training programs for clinical staff (69), especially in units caring for young men and individuals under fitness-related assessment or treatment orders.” However, we do not extend the discussion into the challenges of implementing these practices, in order to be mindful of the growing length of the manuscript and our focus on identifying the individual risk factors. |
| **Reviewer #2:** |  |
| The manuscript presents the results of a study investigating retrospectively risk factors for seclusion and restraint in forensic settings. The authors analyzed retrospectively a large sample of forensic admissions and found specific risk factors for both seclusion and restraint, mostly confirming existing data in general psychiatric settings, with added forensic specificities such as legal admission status. | Thank you for your encouraging insights and recommendations for improvement. |
| **General comment:** The study addresses an interesting and understudied topic. Issue of coercion in psychiatric forensic institutions is rarely investigated. However, some methodological issues greatly impair the quality of this work. | We have made a number of methodological improvements in response to suggestions, as detailed below. |
|  |  |
| **Introduction:** The introduction is too long. The writing style is very repetitive and thus difficult to read. Even with this long text, the authors fail to point to the specificities of the forensic context compared to general psychiatric settings. The project’s aims, even if described, are not sufficiently justified. It is unclear why the authors chose this two-step analysis design. | We have reduced the length of the introduction by increasing the focus and reducing the wording and repetition. We have added information on the forensic context as follows: “In Canada, individuals may be detained in a forensic mental health hospital for two main reasons: 1. For short-term evaluation and treatment to assess their fitness to stand trial or determine if they had a mental disorder at the time of the alleged offense, possibly exempting them from criminal responsibility; and 2. For long-term rehabilitation and reintegration after the court finds them unfit to stand trial (UST) or not criminally responsible due to a mental disorder (NCRMD) (41). While some forensic patients may be admitted to units with minimum security (i.e., open or locked door, limited unaccompanied time off unit), many begin their forensic hospitalization under medium security (i.e., locked door with restricted time off unit) or high security (i.e., perimeter security, locked door, restricted movement within the facility).”  We have now forgone the two-step analysis design. Instead, we are now employing a multilevel logistic regression model using a LOGIT link (Generalized Linear Mixed Model – GLMM), which enables us to estimate the strength of associations between coercive measures (seclusion and restraint) and various factors, while accounting for the clustering of responses within facilities/hospitals. |
| **Methods:** The definition of restraint as both mechanical and manual restraint is questionable, as both kinds of restraint generally have different durations and are experienced very differently by patients. | We agree that mechanical and manual restraint may have different impacts; however, they were each present in < 1% of the sample and in order to examine risk factors for any restraint it was necessary to combine them. We now explain in the Variables section: “Restraint was defined as either mechanical restraint, whereby a patient was being restrained in bed or through wrist restraints only (n = 193), or physical restraint, whereby the patient was physically held to restrict their movement for a brief period to restore calm to the individual (n = 167) (56); although mechanical and physical holding are different restraint strategies, they had similar durations (i.e., modal use < daily) and due to the relatively low proportion of the sample, it was necessary to combine these cases to capture all restraint, similar to Hui and colleagues findings (57).”  We also acknowledge in the Limitations section: “. We combined mechanical and physical restraint due to low proportions, even though they may be different strategies and experiences. Future research using time spent in seclusion or different kinds of restraint is required to determine whether the risk factors may differ.” |
| The exact content of the RAI-MH remains very obscure throughout the Methods section. It seems to comprise among other instruments unstandardized scales used to assess clinical characteristics such as insight or compliance. There is no clear explanation as to the content of the RAI-MH or the evaluation process. All used scales must be clearly described with appropriate references, and their items listed | We have expanded the description of the RAI-MH measures and added references where applicable. For example we now state: “The RAI-MH includes a measure to assess a patient's awareness of their mental health issues and contributing factors (52). This item was recorded as full, limited, or no insight, which we categorized as 0 = full or limited insight and 1 = no insight. History of Medication Adherence is a measure to determine if the patient took their medication as prescribed in the month prior to admission. The item was coded as 0 = at least 80% adherent or no medication prescribed, and 1 = less than 80% adherent or adherence unknown. The RAI-MH includes various behavioral symptom measures, including elopement attempts/threats. Elopement occurs when an individual attempts to leave or leaves the unit without staff knowledge or formal discharge (unauthorized leave). Elopement attempts/threats were recorded from (0) not exhibited in the last 3 days to (3) symptoms that occurred every day during the last three-day period (40,52). We coded this item as 0 = not present and 1 = present.” |
| The authors do not explain why they chose to categorize almost all of the independent variables, such as age, which clearly impairs the quality of the analysis. The categorization of diagnoses, although following international classifications, mixes bipolar disorders and other mood disorders, knowing that maniac states clearly represent an important risk factor for coercive measures. | We have improved our categorization of diagnoses by not focusing on primary diagnosis. Instead, individuals are identified as having a diagnosis within each category if any one of up to four recorded diagnoses falls within that category. In the Variables section, we state, “Up to three diagnoses were documented using DSM-IV (n = 2,501) or DSM-5 (n = 5,134). We recoded all diagnoses to indicate whether each individual had a diagnosis within each of six categories: 1 = schizophrenia spectrum and other psychotic disorders, 2 = substance use disorders, 3 = mood or anxiety disorders, 4 = neurocognitive disorders, 5 = personality disorders, 6 = neurodevelopmental disorders.”  We did not separate mood disorders into bipolar and other mood disorders because not all our cases had this level of detail available. We agree with the reviewer that a manic state is important to examine. Therefore, we did include a clinical scale of mania among our other variables. This measures the presence of a manic state more precisely than a diagnosis of bipolar disorder can, so we feel that we have addressed this important risk factor for coercive measures. |
| As to the analysis, authors should again clearly state the hypotheses instead of using numbers. The description of the multivariate logistic regression is insufficient. Authors should justify their decision to use two separate models and clearly describe all variables used in the models and the used method to enter independent variables in the model. | Thank you for bringing these shortcomings to our attention. We have strengthened our aim and objectives, and we now state: “The present study aimed to identify and validate factors associated with seclusion and restraint that can be assessed upon admission, using a large sample of men and women admitted to forensic hospitals in Ontario, Canada. Specifically, we examined the relative contributions of previously established demographic and clinical risk factors, as well as inconclusive or understudied factors, in predicting the use of coercive measures within the first three days of admission. We hypothesized that younger age, diagnoses of psychotic disorder, substance use disorder, and personality disorder, along with violent and aggressive behavior, would be significant predictors. Additionally, we examined the role of additional demographic (male sex, immigration background, Indigeneity), administrative (legal status), and clinical factors (elopement, lack of insight, medication non-adherence, mania, and cognitive impairment), hypothesizing that these would also contribute to the likelihood of seclusion and restraint in forensic hospitals.”  In our data analysis section we now state: “We conducted unadjusted binary logistic regression analyses with independent variables to examine individual association with seclusion and restraint. To account for the clustering of responses within facilities/hospitals, we employed logistic Generalized Linear Mixed Model (GLMM). This approach allowed us to estimate the strength of the association between coercive measures (seclusion and restraint) and patient-level factors, including sociodemographic, administrative, and clinical factors, accounting for clustering within hospitals. We analyzed seclusion and restraint separately. ” |
| **Results:** The Results section is, as the introduction, too long and difficult to read. There are way too many duplicates between text and tables. | We have carefully avoided duplicating the results reported in the text and the tables. This, along with other careful editing, has reduced the length of the Results level and, we hope, improved ease of reading. |
| The authors keep comparing results of the bivariate analysis and of the multivariate regression, whereas only the multivariate analysis should be extensively considered for discussion. There is no clear added value of using both regression models, as this renders the interpretation of results very confuse | We have kept the bivariate analysis for comparability with previous studies, but we now focus our results and discussion on the multivariate analysis. Further, we have combined the previous two stage regression models into a one-step single model for seclusion and a one-step single model for restraint |
| **Discussion:** As in the Results section, the authors systematically compare bivariate and multivariate analysis, which does not add any substantial information for the understanding of the study results. They also fail to clearly provide thorough explanation of the observed risk factors, and to draw clinical consequences of the analysis. The specificities of the forensic setting could be more clearly describe. The different legal statuses for example are difficult to understand for the non-expert reader. | We have minimized our discussion of the bivariate analysis and now focus primarily on the results logistic GLMM models. We now provide a more thorough discussion of the reasoning behind the associations between risk factors and restraint and seclusion. We further elaborated on the clinical implications and improved our description of the forensic setting, especially the implications of the different legal statuses. For example, we now state: “The present study confirms some previously identified risk factors for coercive measures in forensic psychiatry. For example, young adults were more likely secluded and restrained than older adults, similar to earlier findings (7,42), despite the present sample’s mean age being higher than in previous studies (6). Aggressive and violent behaviors were significantly associated with higher odds of both restraint and seclusion, underscoring research in which actual and threats of violence are cited as reasons for initiating seclusion and restraint events (6,35,40,43). In addition, being male was a predictive factor for seclusion, adding to evidence from some recent studies (42,43) in contrast to older studies (6). Sex did not contribute to the odds of being restrained in the adjusted logistic GLMM model. This finding differs from Beghi’s and colleagues’ findings for general psychiatric patients in which males were more likely than females to be restrained (35). This difference could be attributable to the small percentage of women in forensic psychiatry (16%) compared to an almost 50:50 split in general psychiatry and only 58 female patients being restrained in the present study, less than 1% of the sample.  The present study offers new insights into previously understudied or equivocal demographic, administrative, and clinical factors associated with seclusion and restraint within the first three days of admission to forensic hospitals. Similar risk factors were identified for both seclusion and restraint. Having an immigration background was not a predictor of seclusion or restraint in our adjusted logistic GLMM models. This result differs from Hansen and colleagues (2020) and Hui and colleagues (2015), who found non-significant positive associations with race and ethnicity in their reviews (6,43). However, the variable in our dataset included any immigration to Canada at any time in the patient’s life, and might not represent racial or ethnic minority individuals. We also examined Indigenous self-identification and found it, too, was not associated with coercive measures in either of the models. This finding contributes to the ongoing discussion about the differences in care between forensic hospitals and correctional institutions, where individuals identifying as Indigenous are at higher risk of seclusion (50,64)….. “ |
| The limitations are insufficiently described and should include some of the elements pointed above. | We have added to the limitations as follows:  “We conducted a secondary analysis of anonymized health records using each variable's available data and existing operational definitions. For example, we were unable to identify diagnoses of bipolar disorder specifically in many cases; however, we mitigated this by including a measure of manic state, an important risk factor for aggression that may be related to restraint and seclusion.”  “We combined mechanical and physical restraint due to low proportions, even though they may be different strategies and experiences. Future research using time spent in seclusion or different kinds of restraint is required to determine whether the risk factors may differ.” |
| The implications are too vague and should also include clear clinical or organizational recommendations. | As detailed in a response to a previous comment, we have substantially expanded on the clinical implications and provided some clear and specific suggestions for practice. We now state: “The present findings provide some evidence to support the practice of individual-level assessments and individualized interventions to reduce the risk of seclusion and restraint. Understanding the factors associated with restraint and seclusion within the first three days of forensic admission allows clinicians to identify risk factors in the crucial time period shortly after a patient is admitted and provide timely interventions to limit the onset of the use of coercive measures. Our findings regarding clinical risk factors suggest valuable interventions among at-risk forensic in-patients including evidence-based pharmacological and psychosocial treatments. In particular, evidence-based interventions for the prevention of in-hospital aggression are indicated by our findings and may include both violence risk assessment (68) and assessments specifically to identify individuals at risk of experiencing coercive measures (5), along with aggression management training programs for clinical staff (69), especially in units caring for young men and individuals under fitness-related assessment or treatment orders. “ |
