# Supplementary material for: Factors associated with seclusion and restraint on admission to forensic psychiatric hospitals: A 10-year retrospective study
Source: PLoS One. 2025 Jul 23;20(7):e0328164. doi: 10.1371/journal.pone.0328164 (PMC12286363; doi:10.1371/journal.pone.0328164)
Supplement: Attachment — Submitted filename: Response to editor and reviewers, 2nd revision.docx [file pone.0328164.s004.docx]

Zelalem Belayneh
Academic Editor
PLOS ONE

June 6, 2025

Re: revision for PONE-D-24-34524R1

Factors associated with seclusion and restraint on admission to forensic psychiatric hospitals: A 10-year retrospective study

Dear Dr. Zelalem Belayneh,

We sincerely thank you and the reviewers for your time and insightful feedback on our revised paper. Your comments have been invaluable in improving the current version, and we have made every effort to address them. We have clarified the terminologies and conceptual boundaries of our definitions in the limitations section, responded to the reviewers' concerns regarding missing data, and provided further elaboration on the findings related to elopement in the adjusted GLMM model in the discussion.

Please see our point-by-point responses in the table below.

| Comments: Editor | Response 2^nd^ revision |
| --- | --- |
| I have one comment regarding the use of secondary data sources. Existing literature documents inconsistencies in the terminologies and conceptual boundaries used to define coercive or restrictive practices e.g., ([https://www.sciencedirect.com/science/article/abs/pii/S1359178924000168](https://track.editorialmanager.com/CL0/https:%2F%2Fwww.sciencedirect.com%2Fscience%2Farticle%2Fabs%2Fpii%2FS1359178924000168/1/010f01971315de54-dc422121-5e61-4c3b-b3a7-356eebc2afc8-000000/y27DcVidqZQniVDpPyvPe1hj9aT1VVAM8HUYaY0HAtE=213) ) and ([file:///C:/Users/Zmul0002/Downloads/s00127-024-02739-6%20(4).pdf](http://track.editorialmanager.com/CL0/file:%2F%2F%2FC:%2FUsers%2FZmul0002%2FDownloads%2Fs00127-024-02739-6%2520(4).pdf/1/010f01971315de54-dc422121-5e61-4c3b-b3a7-356eebc2afc8-000000/jH4-Kjf1_Puy2rveRH6TUVUYuucZ_geUpGjVU4wbP64=213) ).  These definitional inconsistencies may lead to measurement bias, as interpretations of what constitutes a coercive can vary based on clinicians’ subjective judgments ([https://onlinelibrary.wiley.com/doi/10.1111/jocn.17588](https://track.editorialmanager.com/CL0/https:%2F%2Fonlinelibrary.wiley.com%2Fdoi%2F10.1111%2Fjocn.17588/1/010f01971315de54-dc422121-5e61-4c3b-b3a7-356eebc2afc8-000000/xZlqvm1Yvnox5UZdQRMEOgqX1cQAfLQ_EuzEc85i6M8=213) ). For example, Savage et al ([https://pubmed.ncbi.nlm.nih.gov/38205597/](https://track.editorialmanager.com/CL0/https:%2F%2Fpubmed.ncbi.nlm.nih.gov%2F38205597%2F/1/010f01971315de54-dc422121-5e61-4c3b-b3a7-356eebc2afc8-000000/xrJ4lESWAOo02dMiq-pNxYoHeXhmJn_ljgKfCPwBnkQ=213) ) noted that variation in the types and numbers of practices reported across studies can significantly impact the reported prevalence rates of coercive practices Such inconsistencies could further confound the measurement outcomes given that you have utilized secondary data sources.  I suggest that you address these issues in the discussion section, particularly under limitations, to acknowledge how definitional and reporting variability might affect the interpretation of your findings. | Thank you for bringing this issue regarding inconsistencies in the terminologies and conceptual boundaries to our attention and for your recommendation. We now state in the limitations: *We acknowledge that our definitions of coercive and restrictive practices may affect prevalence rates and that variability in definitions and reporting in the literature could influence the interpretation of risk factors for such practices. For example, we included only physical restraint (hands-on contact) or mechanical restraint (use of physical devices to restrict movement) in our definition of restraint, whereas Belayneh et al., 2024 included chemical restraint (medications intended to inhibit an individual's movement or behavior). Chemical restraint was excluded in our study due to challenges in defining and identifying its use. PRN medications may serve multiple purposes, and clinical records often lack clear documentation of intent. Definitions also vary widely across settings, limiting standardization. To ensure consistency, we used a more conservative definition focused on physical and mechanical restraint. Nonetheless, it is possible that certain forms of restraint are systematically more likely to be applied to patients with specific characteristics. For example, Nicholls et al (2009) reported that men were more likely to receive PRN antipsychotic medication than women, who were more likely to be placed in seclusion. Our exclusion of chemical restraint may therefore affect our finding that gender was not a risk factor for restraint.* |
|  |  |
|  |  |
| Reviewer #2: The authors have made substantial changes to the manuscript. The readability has been improved. The new methodological approach has also improved the quality of the analysis and the results. The introduction is way more informative than before, and the discussion now addresses most of the relevant findings.  Minor revisions:  - Could the authors give information about the number of incomplete datasets that were excluded from analysis?  - The aspect of elopement behaviour could be discussed some more, as the findings might seem surprising from a general psychiatric point of view. | We are pleased that we were able to improve the readability and quality of analysis and results in our prior revision, and that the introduction and discussion are now more informative and streamlined. Regarding missing data, the dataset was complete with no missing values. As noted in the data sources section, all variables were mandatory reporting requirements for Ontario hospitals and had to be completed within 72 hours of admission to a mental health bed. This ensured a fully complete dataset, with no concerns related to missingness in the analysis.  Regarding your suggestion to elaborate on the aspect of elopement behaviour, the Ontario Mental Health Reporting System Resource Manual, 2017–2018 defines elopement attempts/threats as: The person attempts to leave, or actually leaves, the unit without the staff’s knowledge and/or formal discharge (e.g., unauthorized leave of absence). This includes the intent to abscond without leave for any reason, as well as occasions when the person threatens to leave the unit, making him or her an elopement risk. We now state in the discussion:  *The adjusted model accounted for aggression, violence, mania, and cognitive impairment, all of which were strong predictors of seclusion. This suggests that when these higher-risk behaviors are present, they may overshadow elopement as a trigger for seclusion, or that elopement may co-occur with less severe clinical presentations. Additionally, staff may respond to elopement attempts with enhanced observation or environmental controls rather than seclusion, particularly in settings where escape is unlikely due to secure infrastructure. It is also possible that elopement behavior in this context reflects a form of goal-directed, non-aggressive behavior that staff do not perceive as warranting seclusion, especially if it is not accompanied by violence or acute agitation. Finally, the temporal focus on the first three days of admission may capture a period when staff are particularly vigilant and may use alternative strategies to manage risk, further reducing reliance on seclusion for elopement attempts. Thus, the inverse association observed in the adjusted model likely reflects a complex interplay between patient behavior, staff response, and the secure forensic environment, rather than a simple causal relationship.* |
